# Integrated Chinese Herbal Medicine Therapy Improves the Survival of Patients With Ovarian Cancer

**DOI:** 10.1177/1534735419881497

**Published:** 2019-12-09

**Authors:** Cherry Yin-Yi Chang, Pei-Yuu Yang, Fuu-Jen Tsai, Te-Mao Li, Jian-Shiun Chiou, Chao-Jung Chen, Ting-Hsu Lin, Chiu-Chu Liao, Shao-Mei Huang, Bo Ban, Wen-Miin Liang, Ying-Ju Lin

**Affiliations:** 1 China Medical University Hospital, Taichung; 2 Taipei Medical University Hospital, Taipei; 3 China Medical University, Taichung; 4 Asia University, Taichung; 5 Chinese Research Center for Behavior Medicine in Growth and Development, Jining, Shandong, China

**Keywords:** ovarian cancer, Chinese herbal medicine, overall survival rate, association rule, network analysis

## Abstract

**Background:** Ovarian cancer is the seventh most commonly diagnosed malignancy worldwide and has the highest mortality rate among all gynecological cancers. Chinese herbal medicine (CHM) is widely applied in Taiwan and has been used in integrated therapies to treat patients with cancer. **Methods:** Patients with ovarian cancer who were registered in the Taiwan Registry for Catastrophic Illness Patients Database between 1997 and 2012 were considered for this study. A 1:1 individual matching by age was implemented. A total of 101 CHM users and 101 non-CHM users were involved. A Cox proportional hazard regression model was applied to evaluate the hazard ratio of overall mortality. The Kaplan-Meier method and log-rank test were used to calculate the cumulative incidence of the overall survival rate. Association rule mining and network analysis were used to analyze CHM prescription patterns. **Results:** CHM users showed a significantly lower risk of overall mortality than nonusers (hazard ratio = 0.45, 95% confidence interval = 0.23-0.91; *P* = .0256; multivariate Cox proportional hazard model). The cumulative incidence of the overall survival probability was higher for CHM users than for non-CHM users (log-rank test, *P* = .0009). Association rule mining and network analysis suggested that the main CHM cluster was associated with the usage of Bu-Zhong-Yi-Qi-Tang, Chuan-Xiong, and Xi-Xin, followed by the use of Bai-Shao, Da-Huang, and Di-Huang. **Conclusions:** CHM, as an adjunctive therapy, may reduce the overall mortality in patients with ovarian cancer. A list of herbal medicines that could potentially be used in future studies and clinical trials has also been provided.

## Introduction

Ovarian cancer is the second most common gynecological malignancy in developed countries and has the highest mortality rate among gynecological cancers.^1,2^ Early detection and diagnosis are difficult owing to the lack of obvious symptoms; therefore, patients are usually diagnosed at advanced stages. In Taiwan, 43% of patients with ovarian cancer are diagnosed with high-grade serous carcinoma.^3,4^ The overall survival rate in patients with ovarian cancer is variable and associated with the stage at diagnosis.^5-7^

The therapeutic approach for ovarian cancer is an integration of staging/debulking surgery and chemotherapy.^1,8^ However, 75% of patients experience tumor recurrence after surgery. Other conventional treatments include radiotherapy.^9^ Patients may receive a combination of regimens of chemotherapy and/or radiotherapy after surgical excision to prevent the recurrence of ovarian cancer. However, owing to the heterogeneous histological types of ovarian cancer with various molecular, clinical, and pathological characteristics, personalized medicine may be needed to improve the overall survival rate in patients.^4^

Platinum-based chemotherapies (usually cisplatin or carboplatin) are effective treatments for ovarian cancer.^10,11^ Taxane-based chemotherapy (paclitaxel) is administered along with platinum-based chemotherapy to improve response and/or overcome resistance development.^12^ However, recurrence still occurs and patients develop resistance to chemotherapy. There is a need to develop better therapeutic agents for use alone or in combination with conventional chemotherapies. In Taiwan, Chinese herbal medicine (CHM) has been widely used in integrated therapies for cancer patients, including those with breast, stomach, liver, lung, pancreatic, and prostate cancers.^13-19^ In addition, CHMs or their related natural compounds exhibit beneficial effects against ovarian cancer by enhancing cancer cell apoptosis, reducing multidrug resistance, or alleviating complications of regular treatment.^20-23^

Thus far, there have been no clinical treatments using CHM for patients with ovarian cancer; therefore, its clinical efficacy and overall survival rate need to be investigated. The association between CHM prescription patterns with increased survival rates in patients with ovarian cancer remains to be elucidated. Moreover, the effect of long-term use of CHM in patients with ovarian cancer needs to be studied. Therefore, a population-based database was utilized to investigate the demographic characteristics, cumulative incidences of overall mortality, and CHM prescription patterns for patients with ovarian cancer in Taiwan.

## Methods

### Data Source

The data source used in this study was obtained from the National Health Insurance Research database (NHIRD; http://nhird.nhri.org.tw/) of the National Health Insurance (NHI) program (https://www.nhi.gov.tw/english/) and maintained and managed by the National Health Research Institute in Taiwan. The Registry for Catastrophic Illness Patients was part of the NHIRD and was used to identify patients with cancer involved in this study. The Registry for Catastrophic Illness Patients registers patients, including patients with cancer, noting their pathological diagnoses, and laboratory and clinical examination histories. This was approved by the NHI administration. Patients with catastrophic illness certificates had reduced or free copayments.

Information from these data source included age, sex, symptoms, diagnosis of disease, prescriptions, procedures, records of clinical visits and hospitalization, inpatient orders, ambulatory care, and sociodemographic factors. The International Classification of Diseases, Ninth Revision, Clinical Modification (ICD-9-CM) was used to identify the study population. These medical records were anonymized, and informed consent was not required. This study was designed as a longitudinal and retrospective cohort study and was approved by the Institutional Review Board of China Medical University Hospital, Taiwan (Approval Number: CMUH107-REC3-074(AR-1).

### Study Population

**Figure 1.**
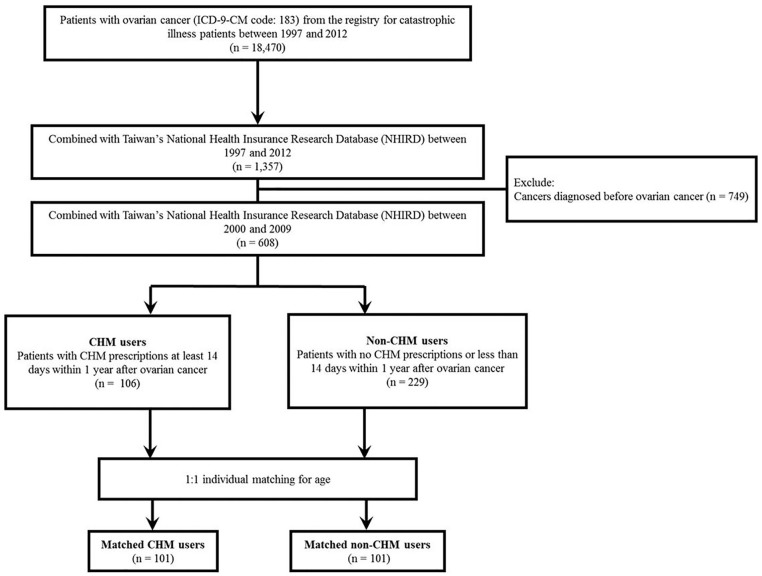
Flowchart for the selection of study participants. Abbreviations: ICD-9-CM, International Classification of Diseases, Ninth Revision, Clinical Modification; CHM, Chinese herbal medicine.

A total of 18 470 patients with ovarian cancer (ICD-9-CM: 183) were selected from the Registry for Catastrophic Illness Patients between 1997 and 2012 (Figure 1). This information was then combined with Taiwan’s NHIRD between 1997 and 2012, which resulted in the selection of 1357 patients with ovarian cancer. Patients with ovarian cancer between 2000 and 2009 who were followed-up until the end of this study were further selected, thereby including 608 patients with ovarian cancer from Taiwan (Figure 1 and Figure S2 [available online]).

From these patients, CHM users (n = 106) were defined as patients with at least 14 cumulative CHM treatment days within the first year after ovarian cancer diagnosis (Figure S1A). Non-CHM users were defined as patients who did not receive any CHM or received CHM treatments for a cumulative period of <14 days within 1 year after ovarian cancer diagnosis. Furthermore, patients who received CHM treatments for >30 days during the entire study period were excluded from the non-CHM group (n = 229). The index date for the CHM users was defined as the date of completion of the 14 days of CHM treatment (Figure S1A). The index duration was defined as the duration between the diagnosed date and the index date (Figure S1A-C).

Individual matching between CHM and non-CHM groups with a 1:1 ratio by age was used to reduce the potential bias. The index date for the non-CHM users was defined as the diagnosed date plus the index duration for the corresponding matched CHM user. Consequently, 101 CHM users and 101 non-CHM users were selected as the study population (Figure 1 and Table 1). These 2 groups were then monitored from the index date to the date of death, the date of withdrawal from the NHI program, or the end date of the database (December 31, 2012) (Table S7).

**Table 1.** Demographic Characteristics of Ovarian Cancer Patients According to Chinese Herbal Medicine Usage in Taiwan.

Variable	Total Subjects		*P* ^a^	Matched Subjects		*P* ^a^
	CHM Users (N = 106), n (%)	Non-CHM Users (N = 229), n (%)		CHM Users (N = 101), n (%)	Non-CHM Users (N = 101), n (%)	
Age (mean ± SD)	46.55 ± 13.9	51.57 ± 17.11	***.005*** ^b^	46.91 ± 13.64	46.85 ± 13.13	.975
Duration from ovarian cancer to the index date (day)^c^	ND	ND	ND	160.46 ± 103.85	160.46 ± 103.85	1.000
Comorbidities^d^						
Hypertension	19 (17.92%)	54 (23.58%)	.243	19 (18.81%)	27 (26.73%)	.180
Diabetes	9 (8.49%)	26 (11.35%)	.426	9 (8.91%)	8 (7.92%)	.800
Hyperlipidemia	12 (11.32%)	26 (11.35%)	.993	12 (11.88%)	9 (8.91%)	.489
Cardiovascular diseases	32 (30.19%)	81 (35.37%)	.351	32 (31.68%)	38 (37.62%)	.375
Treatment method						
Chemotherapy	78 (73.58%)	144 (62.88%)	.054	75 (74.26%)	63 (62.38%)	.070
Radiotherapy	2 (1.89%)	1 (0.44%)	.190	2 (1.98%)	1 (0.99%)	.561
Surgery	79 (74.53%)	179 (78.17%)	.462	76 (75.25%)	79 (78.22%)	.617
Income			.063			.473
<NT20 000	41 (42.27%)	100 (52.08%)		38 (40.86%)	42 (48.28%)	
NT20 000-NT30 000	23 (23.71%)	51 (26.56%)		23 (24.73%)	22 (25.29%)	
≥NT30 000	33 (34.02%)	41 (21.35%)		32 (34.41%)	23 (26.44%)	
Urbanization level^e^			.774			.712
1	41 (39.42%)	86 (39.45%)		39 (39.39%)	34 (35.79%)	
2	29 (27.88%)	68 (31.19%)		27 (27.27%)	31 (32.63%)	
3	34 (32.69%)	64 (29.36%)		33 (33.33%)	30 (31.58%)	

Abbreviations: CHM, Chinese herbal medicine; ND, not determined; NT, new Taiwan dollar; ICD-9-CM, International Classification of Diseases, Ninth Revision, Clinical Modification.

a *P* values were obtained by χ^2^ test.

b *P* < .05 is highlighted in bold italic.

c The duration was defined between the diagnosed date of ovarian cancer and the index date. The index date for the CHM users were defined as the date of completion of the 14 days of treatment of CHM.

d The comorbidities include hypertension (ICD-9-CM: 401-405), diabetes (ICD-9-CM: 250), hyperlipidemia (ICD-9-CM: 272), and cardiovascular diseases (ICD-9-CM: 390-459). These comorbidities and treatment history were recorded before the index date. The index date was defined as the date on which the CHM treatment schedule was completed. Individual matching method was performed for age.

e Urbanization level 1 indicates the highest level; urbanization level 3 indicates the lowest level.

### Chinese Herbal Medicine

Herbal formulas and single herbs were the 2 major forms of CHM used in this study (Table S1). The codes for herbal formulas and single herbs were collected, grouped, and listed on the Taiwan NHI website (http://www.nhi.gov.tw/webdata/webdata.aspx?menu=21&menu_id=713&webdata_id=932). Herbal formulas were composed of a combination of 2 or more herbs provided by a traditional Chinese medicine (TCM) practitioner based on TCM or ancient medical books (Table S1). Single herbs were from plant, animal, or mineral sources. The CHM products in Taiwan are all manufactured by pharmaceutical manufacturers with Good Manufacturing Practice certification. The main pharmaceutical manufacturers were Sun Ten Pharmaceutical Co Ltd (http://www.sunten.com.tw/), Chuang Song Zong Pharmaceutical Co Ltd (http://www.csz.com.tw/), Sheng Chang Pharmaceutical Co Ltd (http://www.herb.com.tw/about_en.php), KO DA Pharmaceutical Co Ltd (http://www.koda.com.tw/), and Kaiser Pharmaceutical Co Ltd (http://www.kpc.com/).

### Association Rule and Network Analysis

The CHM prescription patterns were analyzed by association rule and network analysis. Association rule mining was calculated using the arules_1.6 package of R software (version 3.4.3). The support value (%) was defined as (the frequency of prescriptions of CHM_X and CHM_Y products/total prescriptions) × 100%. The confidence value (CHM_X → CHM_Y; %) was defined as (the frequency of prescriptions of CHM_X and CHM_Y products/frequency of prescriptions of CHM_X product) × 100%. P(Y) (%) means (frequency of prescriptions of Y product/total prescriptions) × 100%. The lift value gave the confidence (CHM_X → CHM_Y) (%)/P(Y) (%). Cytoscape (https://cytoscape.org/, version 3.7.0) was employed to analyze the core prescription pattern of the CHM network for patients with ovarian cancer.

### Study Variables and Treatment History

Study variables included age, comorbidities, treatment history, income, and urbanization level (Table 1 and Tables S3 and S4). Age was expressed as a continuous variable. Comorbidities, treatment history, income, and urbanization levels were recorded before the index date and were expressed as category variables. Comorbidities included hypertension (ICD-9-CM: 401-405), diabetes (ICD-9-CM: 250), hyperlipidemia (ICD-9-CM: 272), and cardiovascular diseases (ICD-9-CM: 390-459). Treatment history included chemotherapy (ATC code: L01; antineoplastic agents), radiotherapy (ICD-9-CM: 183 and OP codes: 36001B, 36001BA, 36015B, 36002B, 36004B, 36005B, 36006B, 36009B, 36010B, 36011B, 36012B, 36013B, 36014B, 37024A1, 36018B, 36019B, 36012BC, 36020B, and 36021C), and surgery (ICD-9-CM: 183 and OP codes: 80424B, 80417A, 80417B, 80419A, 80419B, 78011B1, 81021B, 70208B, 70209B, 70207BA, 70212B, 81016B1, and 80809B). Income was divided into 3 subgroups (Table 1; <NT20 000, NT20 000 to NT30 000, and ≥NT30 000). Urbanization levels in Taiwan were divided into 3 subgroups, where level 1 indicated the highest level and level 3 indicated the lowest level.

### Statistical Analysis

**Figure 2.**
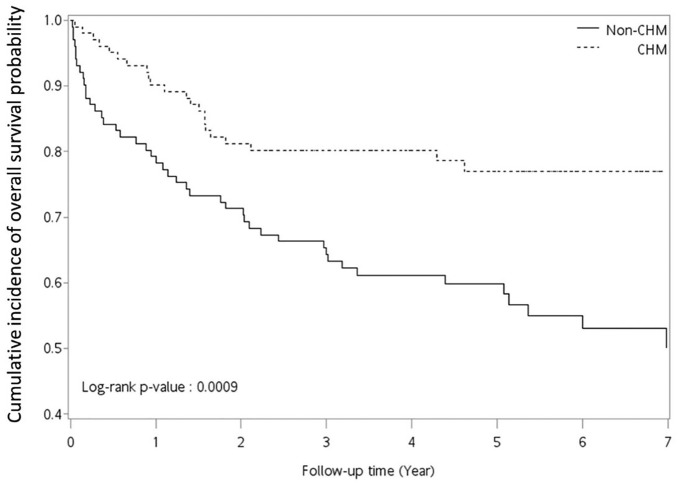
Cumulative incidence of the overall survival probability in patients with ovarian cancer according to Chinese herbal medicine (CHM) usage. CHM (cumulative CHM drug days ≥14 days within 1 year) and their corresponding matched non-CHM users.

Categorical data were expressed as numbers and percentages, including comorbidities, treatments (chemotherapy, radiotherapy, and surgery), income, and urbanization level, and they were assessed using χ^2^ tests. A Cox proportional hazard regression model was applied to evaluate the hazard ratio (HR) and 95% confidence interval (CI) of the risk of overall mortality after adjustment for age, CHM use, comorbidity, and treatment (Table 2 and Tables S5 and S6). The Kaplan-Meier method and the log-rank test were used to calculate the cumulative incidence of overall survival rate according to CHM usage (Figure 2 and Figure S3). All *P* values <.05 were considered to show statistically significant differences. SAS software (version 9.4; SAS Institute, Cary, NC) was used for data management and statistical analyses.

**Table 2.** Hazard Ratios (95% CI) for Overall Mortality in Ovarian Cancer Patients^a,b^.

Variable	Univariate			Multivariate		
	Hazard Ratio	95% CI	*P*	Hazard Ratio	95% CI	*P*
Age	1.05	0.85-1.30	.6600	1.05	0.81-1.35	.7214
CHM use (vs non-CHM use)	0.56	0.32-1.00	.0508	0.45	0.23-0.91	**.0256** ^c^
Comorbidity						
Hypertension (vs no)	0.82	0.34-1.97	.6553	0.93	0.24-3.62	.9213
Diabetes (vs no)	0.33	0.07-1.65	.1785	0.40	0.07-2.43	.3222
Hyperlipidemia (vs no)	1.00	0.29-3.45	1.0000	1.32	0.3-5.93	.7134
Cardiovascular diseases (vs no)	0.89	0.34-2.30	.8087	0.67	0.15-2.9	.5910
Treatment						
Chemotherapy (vs no)	1.67	0.61-4.59	.3226	2.37	0.54-10.42	.2527
Surgery (vs no)	1.50	0.53-4.21	.4417	0.98	0.24-4.03	.9719

Abbreviations: CHM, Chinese herbal medicine; CI, confidence interval.

a Adjusted factors included age, CHM use, comorbidities, and treatments.

b Radiotherapy was excluded (number ≤2; Table 1).

c *p* < 0.05. The value in boldface indicates p < 0.05.

## Results

### Demographic Characteristics

The procedure used to select the study subjects is described in Figure 1. There were 608 patients with ovarian cancer in Taiwan between 2000 and 2009. Among them, 106 patients were assigned as CHM users, as defined by a cumulative period of >14 days of CHM treatment within the first year after diagnosis. There were 229 non-CHM users who received CHM treatment for a cumulative period of <14 days within the first year of diagnosis, and <30 days during the entire study period. As shown in Table 1, there were differences in age. Patients in the non-CHM group were older than those in the CHM group (*P* < .05, Table 1). There were no significant differences in comorbidities, treatment method, income, and urbanization level (*P* > .05, Table 1). To reduce potential bias, 1:1 individual matching by age was performed. As shown, after matching, there were no significant differences between the 2 matched groups.

### Hazard Ratio for Overall Mortality

According to the multivariate Cox proportional hazard model, adjusted for age, CHM use, comorbidity, and treatment, it was found that the CHM users had a lower HR of mortality risk (Table 2; HR = 0.45, 95% CI = 0.23-0.91; *P* = .0256).

Based on the Kaplan-Meier survival curves, the cumulative incidences of overall survival probability were found to be higher for CHM users than non-CHM users (Figure 2; log-rank test, *P* = .0009). These results suggested that CHM as an adjunctive therapy had a protective effect against mortality among patients with ovarian cancer.

### CHM Prescription Pattern

**Figure 3.**
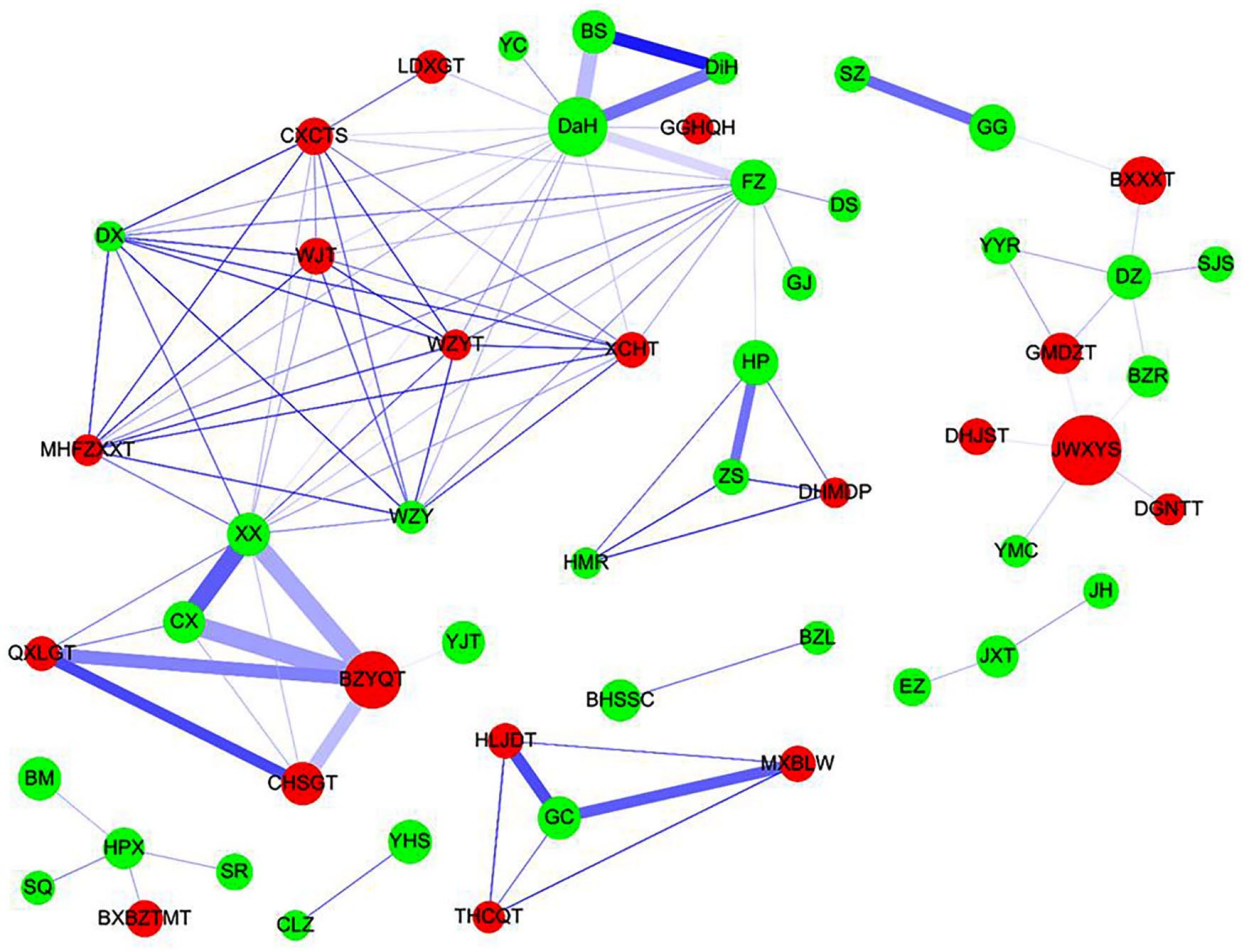
Chinese herbal medicine (CHM) prescription network analysis for patients with ovarian cancer. The lines connecting CHMs represent the support value, where thicker lines represent higher support values and darker lines represent higher lift values. The thicker and darker the connecting line, the more important the connection between CHMs. The size of the circle represents the frequency of prescription of individual CHMs, that is, larger circles represent higher prescription frequencies. Red circles represent herbal formulas and green circles represent single herbs.

Top-ranked CHM prescriptions and their compositions (herbal formula and single herb) by TCM doctors have been listed for use in the treatment of ovarian cancer (Table S1). The top 10 two-CHM combinations were investigated using association rule mining and network analysis (Table 3 and Figure 3). As shown in Table 3, the prescription frequency of 2-CHM combined, support (%), confidence (%), and lift were investigated. Higher values of support, confidence, and lift represented a stronger association between the 2-CHM combinations. The CHM co-prescription pattern Chuan-Xiong (CX) → Bu-Zhong-Yi-Qi-Tang (BZYQT; frequency = 68, support = 2.39%, confidence = 60.18%, and lift = 7.96) had the highest frequency, followed by CX → Xi-Xin (XX; second co-prescription; frequency = 66, support = 2.32%, confidence = 58.41%, and lift = 13.73), and XX → BZYQT (third co-prescription; frequency = 66, support = 2.32%, confidence = 54.55%, and lift = 7.22).

**Table 3.** Ten Most Commonly Used Pairs of CHM Products for Ovarian Cancer Patients in Taiwan.

CHM Products (LHS, X)	Chinese Name		CHM Products (RHS, Y)	Chinese Name	Frequency of Prescriptions of X and Y Products	Support (X) (%)^a^	Confidence (X → Y) (%)^b^	Lift^c,d^
Chuan-Xiong (CX)	川芎	→	Bu-Zhong-Yi-Qi-Tang (BZYQT)	補中益氣湯	68	2.39	60.18	7.96
Chuan-Xiong (CX)	川芎	→	Xi-Xin (XX)	細辛	66	2.32	58.41	13.73
Xi-Xin (XX)	細辛	→	Bu-Zhong-Yi-Qi-Tang (BZYQT)	補中益氣湯	66	2.32	54.55	7.22
Bai-Shao (BS)	白芍	→	Da-Huang (DaH)	大黃	56	1.97	45.90	5.73
Chai-Hu-Shu-Gan-Tang (CHSGT)	柴胡疏肝湯	→	Bu-Zhong-Yi-Qi-Tang (BZYQT)	補中益氣湯	52	1.83	42.98	5.68
Qing-Xin-Li-Ge-Tang (QXLGT)	清心利膈湯	→	Bu-Zhong-Yi-Qi-Tang (BZYQT)	補中益氣湯	51	1.79	78.46	10.38
Di-Huang (DiH)	地黃	→	Da-Huang (DaH)	大黃	48	1.69	94.12	11.74
Di-Huang (DiH)	地黃	→	Bai-Shao (BS)	白芍	48	1.69	94.12	21.94
Huang-Lian-Jie-Du-Tang (HLJDT)	黃連解毒湯	→	Gan-Cao (GC)	甘草	46	1.62	65.71	15.32
Mu-Xiang-Bing-Lang-Wan (MXBLW)	木香檳榔丸	→	Gan-Cao (GC)	甘草	44	1.55	58.67	13.68

Abbreviations: CHM, Chinese herbal medicine; LHS, left-hand side; RHS, right-hand side.

a Support (X) (%) = frequency of prescriptions of X and Y products/total prescriptions × 100%.

b Confidence (X → Y) (%) = frequency of prescriptions of X and Y products/frequency of prescriptions of X product × 100%.

c Lift = Confidence (X → Y) (%)/P(Y) (%).

d P(Y) (%) = frequency of prescriptions of Y product/total prescriptions × 100%.

The CHM network and core treatments prescribed were also investigated (Figure 3). As shown, the CHM combinations and their networks were identified. There was one main CHM cluster with BZYQT, CX, and XX. The second main CHM cluster was Bai-Shao (BS), Da-Huang (DaH), and Di-Huang (DiH).

## Discussion

The study primarily depicts the benefits of CHM as an adjunctive therapy in patients with ovarian cancer. In this retrospective pharmacoepidemiologic study of the use of complementary medicines, it was found that CHM users had lower overall mortality than non-CHM users. The prescription patterns of CHM were investigated using association rule mining and network analysis. The identified CHM products may exhibit protective effects against ovarian cancer and are worthy of future investigations and clinical trials.

There were more patients with higher income in the CHM user group than in the non-CHM user group. However, there were no significant differences in income and urbanization levels between CHM and non-CHM users in total subjects and the matched subjects. It has also been reported previously that leukemia patients who used TCM had a higher sociological situation in Taiwan.^24^ Additionally, stroke patients who used acupuncture had higher income in Taiwan.^25^ Since there were no significant statistical differences in income and urbanization levels between CHM and non-CHM groups in this study, the 2 groups were therefore matched only by age.

Chinese herbal medicine users showed a significantly lower risk of overall mortality than nonusers. These CHM users were those who had at least 14 cumulative days of CHM treatment within the first year after ovarian cancer diagnosis. For the dosages of CHM, 2 additional groups of CHM users were defined. One group of CHM users was defined as patients who had at least 28 cumulative days of CHM treatment within the first year after ovarian cancer diagnosis (Figures S1B and S2A, and Table S3). The second set of CHM users was defined as the patients who had at least 56 cumulative days of CHM treatment within the first year after ovarian cancer diagnosis (Figures S1C and S2B, and Table S4). Both of the Kaplan-Meier survival curves and multivariate Cox proportional hazard model, adjusted for age, CHM use, comorbidity, and treatment, also showed that these 2 additional CHM groups had a lower HR of mortality risk among ovarian cancer patients. These results suggested that those who received CHM as an adjunctive therapy had a protective effect against mortality among patients with ovarian cancer (Figures S3A and S3B, Tables S5 and S6). Complementary CHM therapy also improves survivals of patients with breast, stomach, liver, lung, pancreatic, and prostate cancers.^13-19^

The 5-year survival rate of Asian patients with ovarian cancer was approximately 57% to 64%.^26,27^ In this study in Taiwan, the Kaplan-Meier survival curve also showed that the 5-year survival rate of non-CHM users, in agreement with previous studies, was approximately 60%. The 5-year survival rate of CHM users was approximately 78%. Here, it was observed that patients with ovarian cancer who used CHM as an adjuvant had a higher overall survival probability. The average mean survival time for CHM users was 4.617 years (Table S2). The average mean survival time for non-CHM users was 4.268 years (Table S2). The average median survival time for CHM users was 4.314 years (Table S2). The average median survival time for non-CHM users was 4.186 years (Table S2). Indeed, there are several Chinese herbs and related natural compounds that have anti-metastasis and anti-cell proliferation properties and that induce cancer cell apoptosis and sensitize ovarian cancer cells to chemotherapy.^28-36^ The pharmacoepidemiologic results obtained highlighted the associations between clinically used CHMs and overall mortality in patients with ovarian cancer in Taiwan.

The association rule and network analysis for CHM prescriptions for patients with ovarian cancer suggested that main CHM cluster with BZYQT, CX (*Ligusticum sinense* Oliv.), and XX (*Asarum sieboldii* Miq.) had the highest overall frequencies, support, confidence, and lift. BZYQT is also used for weakness (fatigue, low appetite, or indigestion).^37^ Specifically, BZYQT contains 2 single herbs, Huang-Qi (HQ; *Astragalus membranaceus* [Fisch.] Bunge) and Ren-Shen (RS; *Panax ginseng* C.A.Mey.), which are important in anticancer treatment. Polysaccharides from HQ have been shown to have a chemosensitizing effects on nasopharyngeal carcinoma and melanoma cells.^38,39^ Astragaloside, a natural component of HQ, exhibits anti–ovarian cancer activities.^40^ Ginsenoside-Rb1, a natural compound of RS, targets chemotherapy-resistant ovarian cancer stem cells via simultaneous inhibition of Wnt/β-catenin signaling and epithelial-to-mesenchymal transition.^41^ Ginsenoside Rh-2 exhibits anticancer effects on SKOV3 cells by inhibiting cell proliferation and inducing apoptosis via activation of the caspase-3 and Bcl-2-insensitive pathway.^42^ Ginsenoside Rg3 inhibited the Warburg effect in ovarian cancer cells via the H19/miR-324-5p/PKM2 pathway.^43^ Phthalides and alkaloids are the bioactive ingredients in CX.^44^ 3-Butyldine phthalide and 3-n-butyl phthalide demonstrated cytotoxic activity against human cancer cell lines, including ovarian cancer cells.^45^ Alkaloids are also reported to act as chemosensitizers in ovarian cancer cells via Akt/NF-κB signaling.^46^ Tetramethylpyrazine, a natural compound from CX, inhibits ovarian cell invasion and migration and further exhibits anti-inflammatory properties.^47,48^

The second most common CHM cluster used by patients with ovarian cancer was BS (*Paeonia lactiflora* Pall.), DaH (*Rheum palmatum* L.), and DiH (*Rheum palmatum* L.). Albiflorin and paeoniflorin, the main components of BS, act as an adjunctive drug in cancer treatment by ameliorating side effects induced by radiotherapy and chemotherapy.^49^ There are 14 natural compounds of DaH,^50^ among which gallic acid exhibits antiangiogenic effects via the PTEN/AKT/HIF-1α/VEGF signaling pathway in ovarian cancer cells.^51^ Catechin and (−)-epigallocatechin-3-O-gallate of DaH are produced as micellar nanocomplexes for safe and effective cisplatin nanomedicines for ovarian cancer treatment.^52^ Emodin and chrysophanol exhibit anti-ovarian cancer activities, including proliferation suppression, migration, and invasion.^53-55^ Rhein inhibits the migration of ovarian cancer cells and attenuates multidrug resistance in human ovarian cancer.^56,57^ Catalpol, a natural compound of DiH, is a natural Taq DNA polymerase inhibitor and suppresses proliferation and facilitates apoptosis of ovarian cancer cells.^58,59^

This study reveals that these CHMs show potential anti–ovarian cancer activity. This study also provides a list of CHM candidates and their pharmacological networks that could be used for clinical trials with chemotherapy drugs. However, there are also limitations to this study. Information about behaviors, such as diet, nutrients, exercise, drinking, smoking, blood biochemical data, genetic data, and compliance or adherence to medication use, could not be obtained.

## Conclusions

In this study, among patients with ovarian cancer, CHM users had a significantly lower risk of mortality than non-CHM users. The association rule and network analysis for CHM prescriptions for patients with ovarian cancer suggested that the CHM cluster with BZYQT, CX, and XX had the highest overall frequencies, support, confidence, and lift. Further studies should be performed to optimize the safety and efficacy of CHM usage in such patients.

## Supplemental Material

Supplementary_Materials_08122019 – Supplemental material for Integrated Chinese Herbal Medicine Therapy Improves the Survival of Patients With Ovarian Cancer Click here for additional data file. Supplemental material, Supplementary_Materials_08122019 for Integrated Chinese Herbal Medicine Therapy Improves the Survival of Patients With Ovarian Cancer by Cherry Yin-Yi Chang, Pei-Yuu Yang, Fuu-Jen Tsai, Te-Mao Li, Jian-Shiun Chiou, Chao-Jung Chen, Ting-Hsu Lin, Chiu-Chu Liao, Shao-Mei Huang, Bo Ban, Wen-Miin Liang and Ying-Ju Lin in Integrative Cancer Therapies
