# Supplementary material for: Integrated Chinese Herbal Medicine Therapy Improves the Survival of Patients With Ovarian Cancer
Source: Integr Cancer Ther. 2019 Dec 9;18:1534735419881497. doi: 10.1177/1534735419881497 (PMC6902381; doi:10.1177/1534735419881497)
Supplement: Supplementary_Materials_08122019 – Supplemental material for Integrated Chinese Herbal Medicine Therapy Improves the Survival of Patients With Ovarian Cancer [file Supplementary_Materials_08122019.pdf]

Supplementary Materials

Table S1. Composition of the Most Commonly Used Herbal Formulas and Single Herbs for Patients with Ovarian Cancer in Taiwan.

Table S2. Average Survival Time of Patients with Ovarian Cancer between CHM and non-CHM Users.

Table S3. Demographic Characteristics of Patients with Ovarian Cancer According to (CHM) Usage in Taiwan (CHM (cumulative CHM drug days ≧28 days within 1 year) and their corresponding matched non-CHM users).

Table S4. Demographic Characteristics of Patients with Ovarian Cancer According to (CHM) Usage in Taiwan (CHM (cumulative CHM drug days ≧56 days within 1 year) and their corresponding matched non-CHM users).

Table S5. Hazard Ratios (95% CI) for Overall Mortality in Patients with Ovarian Cancer (CHM (cumulative CHM drug days ≧28 days within 1 year) and their corresponding matched non-CHM users).

Table S6. Hazard Ratios (95% CI) for Overall Mortality in Patients with Ovarian Cancer (CHM (cumulative CHM drug days ≧56 days within 1 year) and their corresponding matched non-CHM users).

Table S7. Distribution of the Cumulative Period of CHM Treatment of CHM Users Among Ovarian Cancer Patients in This Study in Taiwan (from the index date to the study end).

Figure S1. Follow-up time for CHM and matched non-CHM users. **A.** CHM (cumulative CHM drug days ≧ 14 days within 1 year) and their corresponding matched non-CHM users. **B.** CHM (cumulative CHM drug

days ≧28 days within 1 year) and their corresponding matched non-CHM users. **C.** CHM (cumulative CHM drug days ≧56 days within 1 year) and their corresponding matched non-CHM users.

Figure S2. Flowchart for the selection of study participants. **A.** CHM (cumulative CHM drug days ≧28 days within 1 year) and their corresponding matched non-CHM users. **B.** CHM (cumulative CHM drug days ≧56 days within 1 year) and their corresponding matched non-CHM users.

Figure S3. Cumulative incidence of the overall survival probability in patients with ovarian cancer according to CHM usage. **A.** CHM (cumulative CHM drug days ≧28 days within 1 year) and their corresponding matched non-CHM users. **B.** CHM (cumulative CHM drug days ≧56 days within 1 year) and their corresponding matched non-CHM users.

Table S1. Composition of the Most Commonly Used Herbal Formulas and Single Herbs for Patients with Ovarian Cancer in Taiwan.

**Formulas**	**Chinese name**	**Number of herbs**	**Composition (Pin-yin name (latin name; botanical plant name))**	**Frequency of prescriptions**	**Frequency of user**	**Person-year**	**Percentage of usage person**	**Avg. drug dose per day (g)**	**Average duration for prescription (days)**
**Total**				**2844**	**101**	**466.7**	**100**	**12.7**	**8.9**
**Herbal formula (Pin-yin name)**				**2665**	**101**	**466.7**	**100**	**9.6**	**8.9**
Jia-Wei-Xiao-Yao-San (JWXYS)	加味逍遙散	10	**Dang-Gui** (*Radix Angelicae Sinensi*; *Angelica sinensis (Oliv.) Diels*), **Bai-Shao** (*Radix Paeoniae Alba*; *Paeonia lactiflora Pall.*), **Fu-Ling** (*Poria*; *Wolfiporia extensa (Peck) Ginns*), **Bai-Zhu** (*Rhizoma Atractylodis Macrocephalae*; *Atractylodes macrocephala Koidz.*), **Chai-Hu** (*Radix Bupleuri*; *Bupleurum falcatum L.*), **Mu-Dan-Pi** (*Cortex Moutan*; *Moutan officinalis (L.) Lindl. & Paxton*), **Zhi-Zi** (*Fructus Gardeniae*; *Gardenia jasminoides J.Ellis*), **Gan-Cao** (*Radix Glycyrrhizae Preparata*; *Glycyrrhiza uralensis Fisch.*), **Bo-He** (*Herba Menthae Haplocalycis*; *Mentha arvensis L.*), **Sheng-Jiang** (*Rhizoma Zingiberis Recens*; *Zingiber officinale Roscoe*)	300	52	282.1	51.5	4	10.7

Bu-Zhong-Yi-Qi-Tang (BZYQT)	補中益氣湯	8	**Huang-Qi (***Radix Astragali; Astragalus membranaceus (Fisch.) Bunge***), Ren-Shen (***Radix Ginseng; Panax ginseng C.A.Mey.),* **Bai-Zhu** *(Rhizoma Atractylodis; Atractylodes macrocephala Koidz.***)**, **Gan-Cao (***Radix Glycyrrhizae Preparata; Glycyrrhiza uralensis Fisch.***)**, **Dang-Gui (***Radix Angelicae Sinensi; Angelica sinensis (Oliv.) Diels***), Chen-Pi (***Pericarpium Citri Reticulatae; Citrus reticulata Blanco***), Sheng-Ma (***Rhizoma Cimicifugae*; *Cimicifuga foetida L.*), **Chai-Hu** (*Radix Bupleuri; Bupleurum chinense DC.*)	217	32	160.3	31.7	4.7	8.6
Ban-Xia-Xie-Xin-Tang (BXXXT)	半夏瀉心湯	7	**Zhi-Ban-Xia** (*Rhizoma Pinelliae Preparatum; Pinellia ternata (Thunb.) Makino*), **Gan-Jiang** (*Rhizoma Zingiberis; Zingiber acuminatum Valeton*), **Huang-Qin** (Radix Scutellariae; Scutellaria baicalensis Georgi), **Huang-Lian** (*Rhizoma Coptidis; Coptis chinensis Franch.),* **Ren-Shen** *(Radix Ginseng; Panax ginseng C.A.Mey.),* **Da-Zao** *(Fructus Jujube; Ziziphus jujuba Mill.),* **Gan-Cao** *(Radix Glycyrrhizae Preparata; Glycyrrhiza uralensis Fisch.)*	155	16	101	15.8	3.6	10.5
Chai-Hu-Shu-Gan-Tang (CHSGT)	柴胡疏肝湯	7	**Chen-Pi** (*Pericarpium Citri Reticulatae*; *Citrus reticulata Blanco*), **Chai-Hu** (*Radix Bupleuri*; *Bupleurum chinense DC.*), **Chuan-Xiong (***Rhizoma Chuanxiong***;** *Ligusticum sinense Oliv.***), Zhi-Shi (** *Fructus Aurantii Immaturus***;** *Citrus aurantium L.***), Bai-Shao (***Radix Paeoniae Alba***;** *Paeonia lactiflora Pall.***), Zhi-Gan-Cao (** *Radix Glycyrrhizae*	121	17	91.1	16.8	3.2	8

			Preparata; *Glycyrrhiza uralensis Fisch.*), **Xiang-Fu** (*Rhizoma Cyperi*; *Cyperus rotundus L.*)						
Gan-Mai-Da-Zao-Tang (GMDZT)	甘麥大棗湯	3	**Gan-Cao** (*Radix Glycyrrhizae Preparata; Glycyrrhiza uralensis Fisch.*), **Xiao-Mai** (*Fructus Tritici Levis*; *Triticum aestivum L.*), **Da-Zao** (*Fructus Jujube*; *Ziziphus jujuba Mill.*)	109	15	79.2	14.9	3.3	10.2
Chuan-Xiong-Cha-Tiao-San (CXCTS)	川芎茶調散	10	**Bo-He** (*Herba Menthae Haplocalycis*; *Mentha arvensis L.*), **Chuan-Xiong** (*Rhizoma Chuanxiong*; *Ligusticum sinense Oliv.*), **Bai-Zhi** (*Radix Angelicae Dahuricae*; *Angelica dahurica (Hoffm.) Benth. & Hook.f. ex Franch. & Sav.*), **Qiang-Huo** (*Rhizoma seu Radix Notopterygii*; *Notopterygium forbesii var. oviforme (Shan) H.T. Chang*), **Xi-Xin** (*Herba cum Radix Asari*; *Asarum sieboldii Miq.*), **Xiang-Fu** (*Rhizoma Cyperi*; *Cyperus rotundus L.*), **Jing-Jie** (*Herba Schizonepetae*; *Schizonepeta tenuifolia (Benth.) Briq.*), **Fang-Feng** (*Radix Saposhnikoviae*; *Saposhnikovia divaricata (Turcz.) Schischk.*), **Gan-Cao** (*Radix Glycyrrhizae Preparata*; *Glycyrrhiza uralensis Fisch.*), **Lu-Cha** (*Folium Camelliae Sinensis*; *Camellia sinensis (L.) Kuntze*)	83	20	127	19.8	3	6.4
**Single herbs (Pin-yin name)**				**2358**	**98**	**452.6**	**97**	**4.3**	**9.2**
Da-Huang (DaH)	大黃	1	**Da-Huang** (*Radix et Rhizoma Rhei*; *Rheum palmatum L.*)	228	23	114.2	22.8	0.8	8.8

Ge-Gen (GG)	葛根	1	**Ge-Gen** (*Radix Puerariae*; *Pueraria lobata (Willd.) Ohwi*)	144	33	183.3	32.7	1.2	12
Fu-Zi (FZ)	附子	1	**Fu-Zi (*Radix Aconiti Lateralis Preparata*; *Astragalus membranaceus (Fisch.) Bunge*)**	141	23	95.3	22.8	0.7	8
Hou-Po (HP)	厚朴	1	**Hou-Po** (*Cortex Magnoliae Officinalis*; *Magnolia officinalis Rehder & E.H.Wilson*)	135	20	100.9	19.8	1.1	7.8
Du-Zhong (DZ)	杜仲	1	**Du-Zhong** (*Eucommiae cortex*; *Eucommia ulmoides Oliv.*)	129	17	91	16.8	1.3	11.2
Yan-Hu-Suo (YHS)	延胡索	1	**Yan-Hu-Suo** (*Rhizoma Corydalis; Corydalis yanhusuo*)	126	36	192.9	35.6	1.1	8
Bei-Mu (BM)	貝母	1	**Bei-Mu** (*Bulbus Fritillariae Cirrhosae*; *Fritillaria cirrhosa D.Don*)	123	24	147	23.8	0.9	6.6
Bai-Shao (BS)	白芍	1	**Bai-Shao (*Radix Paeoniae Alba*; *Paeonia lactiflora Pall.*)**	122	23	116.4	22.8	1.8	8.2
Gan-Cao (GC)	甘草	1	**Gan-Cao (*Radix Glycyrrhizae Preparata*; *Glycyrrhiza uralensis Fisch.*)**	122	20	108	19.8	1	7.7
Xi-Xin (XX)	細辛	1	**Xi-Xin** (*Herba cum Radix Asari*; *Asarum sieboldii Miq.*)	121	10	65.8	9.9	1	7.4

*Sorted by frequency of prescriptions.

Information are obtained from the websites (http://www.americandragon.com/index.htm; http://old.tcmwiki.com/; http://www.shen-nong.com/eng/front/index.html; http://www.ipni.org/; http://www.theplantlist.org/).

Table S2. Average Survival Time of Patients with Ovarian Cancer between CHM and non-CHM Users.

	Number	Average survival time (mean; years)	Average survival time (median; years)
**Non-CHM users**	**101**	4.268	4.186
**CHM users**	**101**	4.617	4.314

CHM, Chinese herbal medicine.

Table S3. Demographic Characteristics of Patients with Ovarian Cancer According to (CHM) Usage in Taiwan (CHM (cumulative CHM drug days ≧28 days within 1 year) and their corresponding matched non-CHM users).

Variable	Total subjects		*p*-value	Matched subjects		*p*-value
	**CHM users (N=73)**	**Non-CHM users (N=232)**		**CHM users (N=69)**	**Non-CHM users (N=69)**	
	**N (%)**	**N (%)**		**N (%)**	**N (%)**	
**Age (Mean±SD)**	46.61± 13.99	51.61± 17.04	***0.024***	47.14± 13.5	47.38± 12.28	0.915
**Duration from ovarian cancer to the index date (day)**	ND	ND	ND	188.86± 102	188.86± 102	1.000
**Comorbidities**						
Hypertension	16 (21.92%)	55 (23.71%)	0.752	16 (23.19%)	17 (24.64%)	0.842
Diabetes	8 (10.96%)	26 (11.21%)	0.953	8 (11.59%)	6 (8.7%)	0.573
Hyperlipidemia	9 (12.33%)	26 (11.21%)	0.793	9 (13.04%)	7 (10.14%)	0.595
Cardiovascular diseases	27 (36.99%)	82 (35.34%)	0.799	26 (37.68%)	25 (36.23%)	0.860
**Treatment method**						
Chemotherapy	52 (71.23%)	147 (63.36%)	0.218	49 (71.01%)	45 (65.22%)	0.465
Radiotherapy	0 (0.00%)	1 (0.43%)	0.574	0 (0.00%)	0 (0.00%)	-
Surgery	55 (75.34%)	182 (78.45%)	0.578	52 (75.36%)	52 (75.36%)	1.000
**Income**			0.151			0.900
<NT20,000	28 (42.42%)	101 (51.79%)		27 (42.86%)	24 (42.86%)	
NT20,000-NT30,000	16 (24.24%)	52 (26.67%)		16 (25.4%)	16 (28.57%)	
≧NT30,000	22 (33.33%)	42 (21.54%)		20 (31.75%)	16 (28.57%)	
**Urbanization level**			0.528			0.191
1	23 (32.39%)	88 (39.82%)		22 (32.84%)	31 (48.44%)	
2	24 (33.8%)	68 (30.77%)		23 (34.33%)	17 (26.56%)	
3	24 (33.8%)	65 (29.41%)		22 (32.84%)	16 (25%)	

p-values were obtained by chi-square test. *p*-value ($p < 0.05$) was highlighted in bold italic.

CHM, Chinese herbal medicine; N, number; ND, not determined; NT, new Taiwan dollar.

The comorbidities include hypertension (ICD-9-CM: 401-405), diabetes (ICD-9-CM: 250), hyperlipidemia (ICD-9-CM: 272), and cardiovascular diseases (ICD-9-CM: 390-459). These comorbidities and treatment history were recorded before the index date. The index date was defined as the date on which the CHM treatment schedule was completed. Individual matching method was performed for age. Urbanization level 1 indicates the highest level; urbanization level 3 indicates the lowest level.

The duration was defined between the diagnosed date of ovarian cancer and the index date. The index date for the CHM users were defined as the date of completion of the 28 days of treatment of CHM.

Table S4. Demographic Characteristics of Patients with Ovarian Cancer According to (CHM) Usage in Taiwan (CHM (cumulative CHM drug days ≧56 days within 1 year) and their corresponding matched non-CHM users).

Variable	**Total subjects**		***p*-value**	**Matched subjects**		***p*-value**
	**CHM users (N=49)**	**Non-CHM users (N=233)**		**CHM users (N=48)**	**Non-CHM users (N=48)**	
	**N (%)**	**N (%)**		**N (%)**	**N (%)**	
**Age (Mean±SD)**	48.77± 11.72	51.61± 17.01	0.161	49.66± 9.99	49.38± 8.91	0.884
**Duration from ovarian cancer to the index date (day)**	ND	ND	ND	204.42± 89.25	204.42± 89.25	1.000
**Comorbidities**						
Hypertension	11 (22.45%)	55 (23.61%)	0.862	11 (22.92%)	12 (25%)	0.811
Diabetes	4 (8.16%)	26 (11.16%)	0.536	4 (8.33%)	3 (6.25%)	0.695
Hyperlipidemia	5 (10.2%)	26 (11.16%)	0.846	5 (10.42%)	4 (8.33%)	0.726
Cardiovascular diseases	18 (36.73%)	82 (35.19%)	0.838	18 (37.5%)	18 (37.5%)	1.000
**Treatment method**						
Chemotherapy	37 (75.51%)	148 (63.52%)	0.108	37 (77.08%)	36 (75%)	0.811
Radiotherapy	0 (0.00%)	1 (0.43%)	0.646	0 (0.00%)	0 (0.00%)	-
Surgery	39 (79.59%)	183 (78.54%)	0.870	38 (79.17%)	37 (77.08%)	0.805
**Income**			0.150			0.839
<NT20,000	17 (36.96%)	101 (51.79%)		17 (36.96%)	18 (42.86%)	
NT20,000-NT30,000	14 (30.43%)	52 (26.67%)		14 (30.43%)	11 (26.19%)	
≧NT30,000	15 (32.61%)	42 (21.54%)		15 (32.61%)	13 (30.95%)	
**Urbanization level**			0.709			0.334
1	16 (33.33%)	88 (39.64%)		15 (31.91%)	21 (46.67%)	
2	16 (33.33%)	69 (31.08%)		16 (34.04%)	13 (28.89%)	
3	16 (33.33%)	65 (29.28%)		16 (34.04%)	11 (24.44%)	

p-values were obtained by chi-square test. *p*-value ($p < 0.05$) was highlighted in bold italic.

CHM, Chinese herbal medicine; N, number; ND, not determined; NT, new Taiwan dollar.

The comorbidities include hypertension (ICD-9-CM: 401-405), diabetes (ICD-9-CM: 250), hyperlipidemia (ICD-9-CM: 272), and cardiovascular diseases (ICD-9-CM: 390-459). These comorbidities and treatment history were recorded before the index date. The index date was defined as the date on which the CHM treatment schedule was completed. Individual matching method was performed for age. Urbanization level 1 indicates the highest level; urbanization level 3 indicates the lowest level.

The duration was defined between the diagnosed date of ovarian cancer and the index date. The index date for the CHM users were defined as the date of completion of the 56 days of treatment of CHM.

Table S5. Hazard Ratios (95% CI) for Overall Mortality in Patients with Ovarian Cancer (CHM (cumulative CHM drug days ≧28 days within 1 year) and their corresponding matched non-CHM users).

**Variable**	**Univariate**			**Multivariate**		
	**Hazard ratio**	**95% CI**	***p*-value**	**Hazard ratio**	**95% CI**	***p*-value**
**Age**	0.95	(0.74-1.22)	0.6792	0.91	(0.64-1.29)	0.5862
**CHM use (vs. non-CHM use)**	0.44	(0.22-0.89)	***0.0233***	0.32	(0.13-0.78)	***0.0120***
**Comorbidity**						
Hypertension (vs. no)	1.20	(0.37-3.93)	0.7633	4.69	(0.54-40.63)	0.1606
Diabetes (vs. no)	0.75	(0.17-3.35)	0.7064	0.41	(0.06-2.98)	0.3794
Hyperlipidemia (vs. no)	1.00	(0.29-3.45)	1.0000	0.58	(0.08-4.42)	0.5957
Cardiovascular diseases (vs. no)	1.00	(0.38-2.66)	1.0000	0.78	(0.15-4.13)	0.7654
**Treatment**						
Chemotherapy (vs. no)	1.83	(0.68-4.96)	0.2324	2.49	(0.5-12.43)	0.2663
Surgery (vs. no)	1.80	(0.6-5.37)	0.2920	2.15	(0.32-14.68)	0.4333

CHM, Chinese herbal medicine; 95% CI, 95% confidence interval.

Adjusted factors included age, CHM use, comorbidities, and treatments.

Radiotherapy was excluded (number ≤ 2; Table S3).

Table S6. Hazard Ratios (95% CI) for Overall Mortality in Patients with Ovarian Cancer (CHM (cumulative CHM drug days ≧56 days within 1 year) and their corresponding matched non-CHM users).

**Variable**	**Univariate**			**Multivariate**		
	**Hazard ratio**	**95% CI**	***p*-value**	**Hazard ratio**	**95% CI**	***p*-value**
**Age**	1.09	(0.81-1.46)	0.5802	1.13	(0.8-1.6)	0.4833
**CHM use (vs. non-CHM use)**	0.58	(0.28-1.22)	0.1492	0.42	(0.17-1.06)	0.0650
**Comorbidity**						
Hypertension (vs. no)	1.67	(0.4-6.97)	0.4843	0.46	(0.06-3.6)	0.4599
Diabetes (vs. no)	1.00	(0.2-4.96)	1.0000	1.00	(0.13-7.47)	0.9983
Hyperlipidemia (vs. no)	2.50	(0.49-12.89)	0.2734	2.98	(0.43-20.57)	0.2692
Cardiovascular diseases (vs. no)	2.25	(0.69-7.31)	0.1772	4.04	(0.71-23.09)	0.1162
**Treatment**						
Chemotherapy (vs. no)	4.50	(0.97-20.83)	0.0544	ND	ND	ND
Surgery (vs. no)	1.50	(0.42-5.32)	0.5299	ND	ND	ND

CHM, Chinese herbal medicine; 95% CI, 95% confidence interval; ND, not determined.

Adjusted factors included age, CHM use, comorbidities, and treatments.

Radiotherapy was excluded (number ≤ 2; Table S4).

Table S7. Distribution of the Cumulative Period of CHM Treatment of CHM Users Among Ovarian Cancer Patients in This Study in Taiwan (from the index date to the study end).

**Cumulative period of CHM treatment (day)**	**CHM users (N = 101)**	
	**N**	**%**
**<60 day**	30	29.70
**60-120 day**	21	20.79
**120-240 day**	16	15.84
**>240 day**	34	33.66

Figure S1

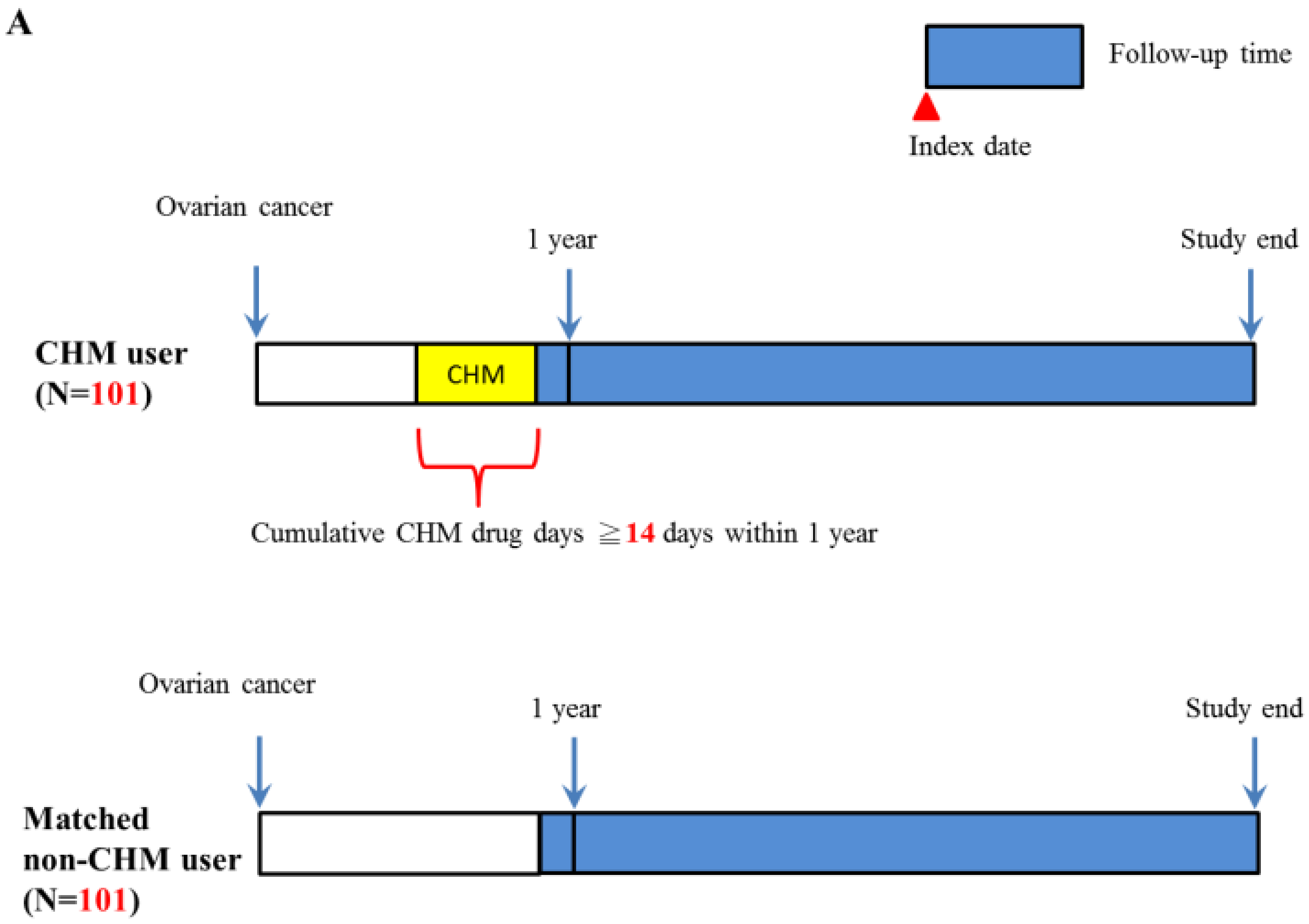
A
Follow-up time
Index date
Ovarian cancer
1 year
Study end
CHM user
(N=101)
CHM
Cumulative CHM drug days ≧14 days within 1 year
Ovarian cancer
1 year
Study end
Matched
non-CHM user
(N=101)

Figure S1

B

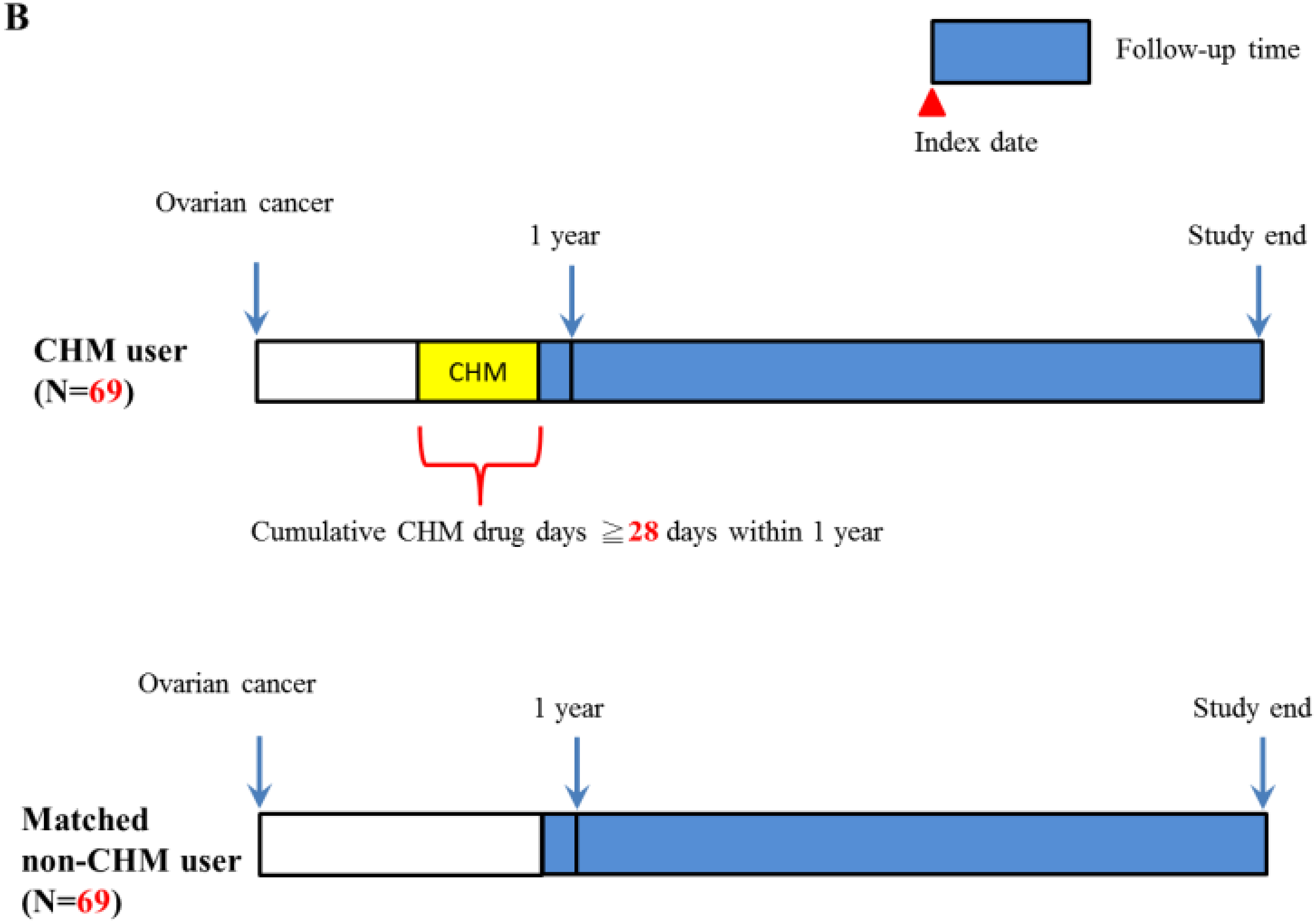
Follow-up time
Index date
Ovarian cancer
1 year
Study end
CHM user
(N=69)
CHM
Cumulative CHM drug days ≧28 days within 1 year
Ovarian cancer
1 year
Study end
Matched
non-CHM user
(N=69)

Figure S1

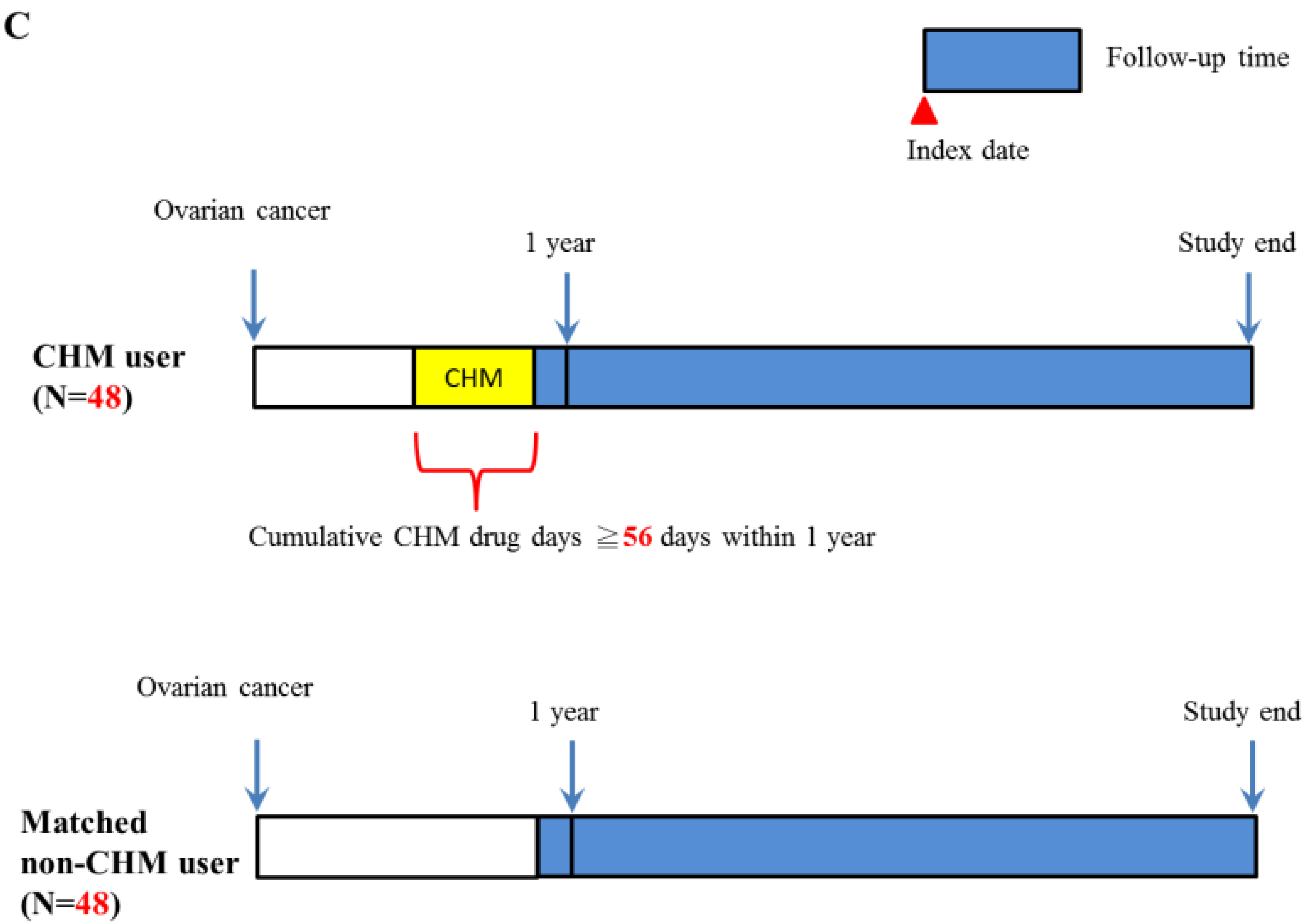
C
Follow-up time
Index date
Ovarian cancer
1 year
Study end
CHM user
(N=48)
CHM
Cumulative CHM drug days ≧56 days within 1 year
Ovarian cancer
1 year
Study end
Matched
non-CHM user
(N=48)

Figure S2

A

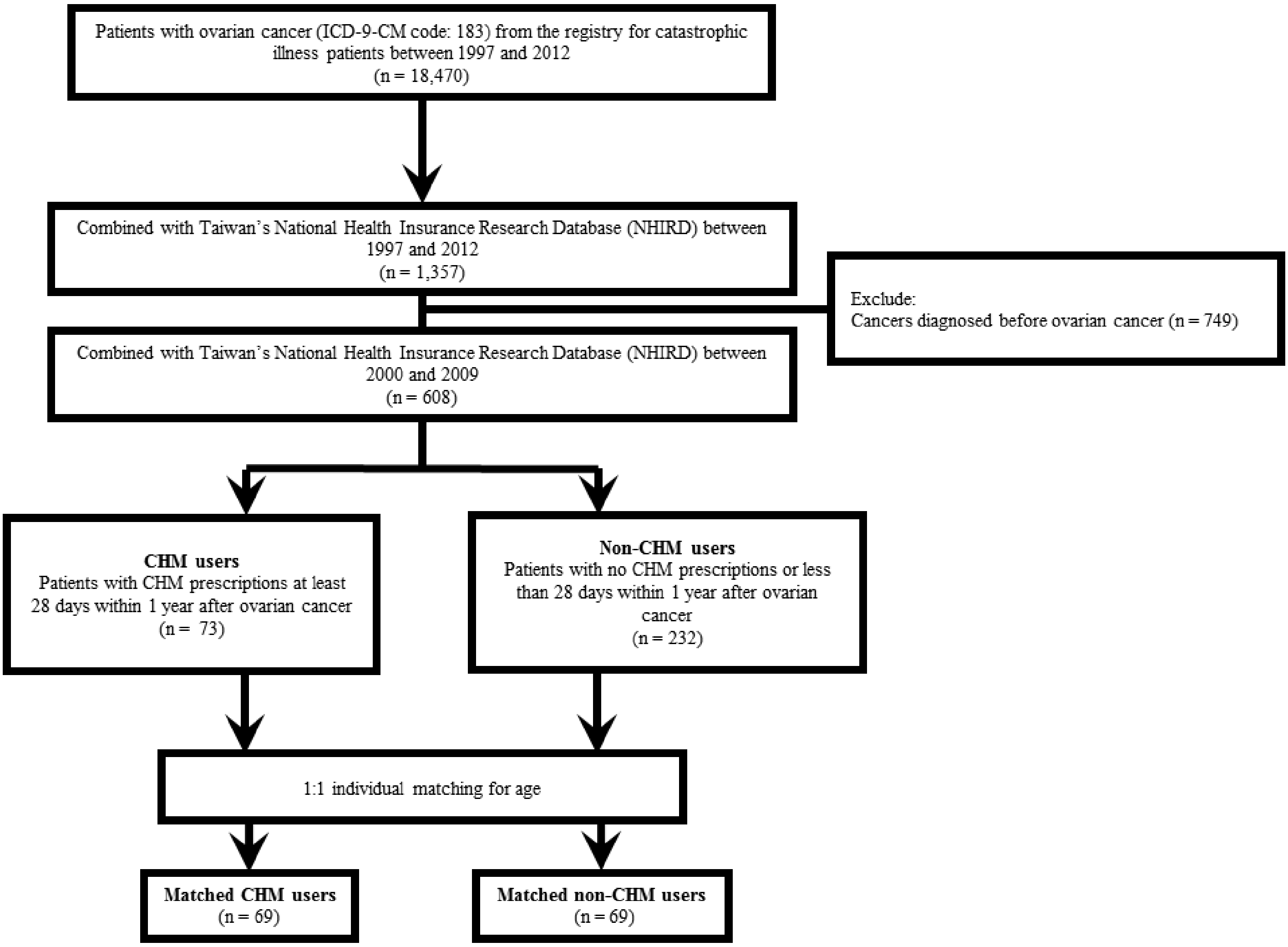

Patients with ovarian cancer (ICD-9-CM code: 183) from the registry for catastrophic illness patients between 1997 and 2012
(n = 18,470)
Combined with Taiwan's National Health Insurance Research Database (NHIRD) between 1997 and 2012
(n = 1,357)
Exclude:
Cancers diagnosed before ovarian cancer (n = 749)
Combined with Taiwan's National Health Insurance Research Database (NHIRD) between 2000 and 2009
(n = 608)
CHM users
Patients with CHM prescriptions at least 28 days within 1 year after ovarian cancer
(n = 73)
Non-CHM users
Patients with no CHM prescriptions or less than 28 days within 1 year after ovarian cancer
(n = 232)
1:1 individual matching for age
Matched CHM users
(n = 69)
Matched non-CHM users
(n = 69)

Figure S2

B

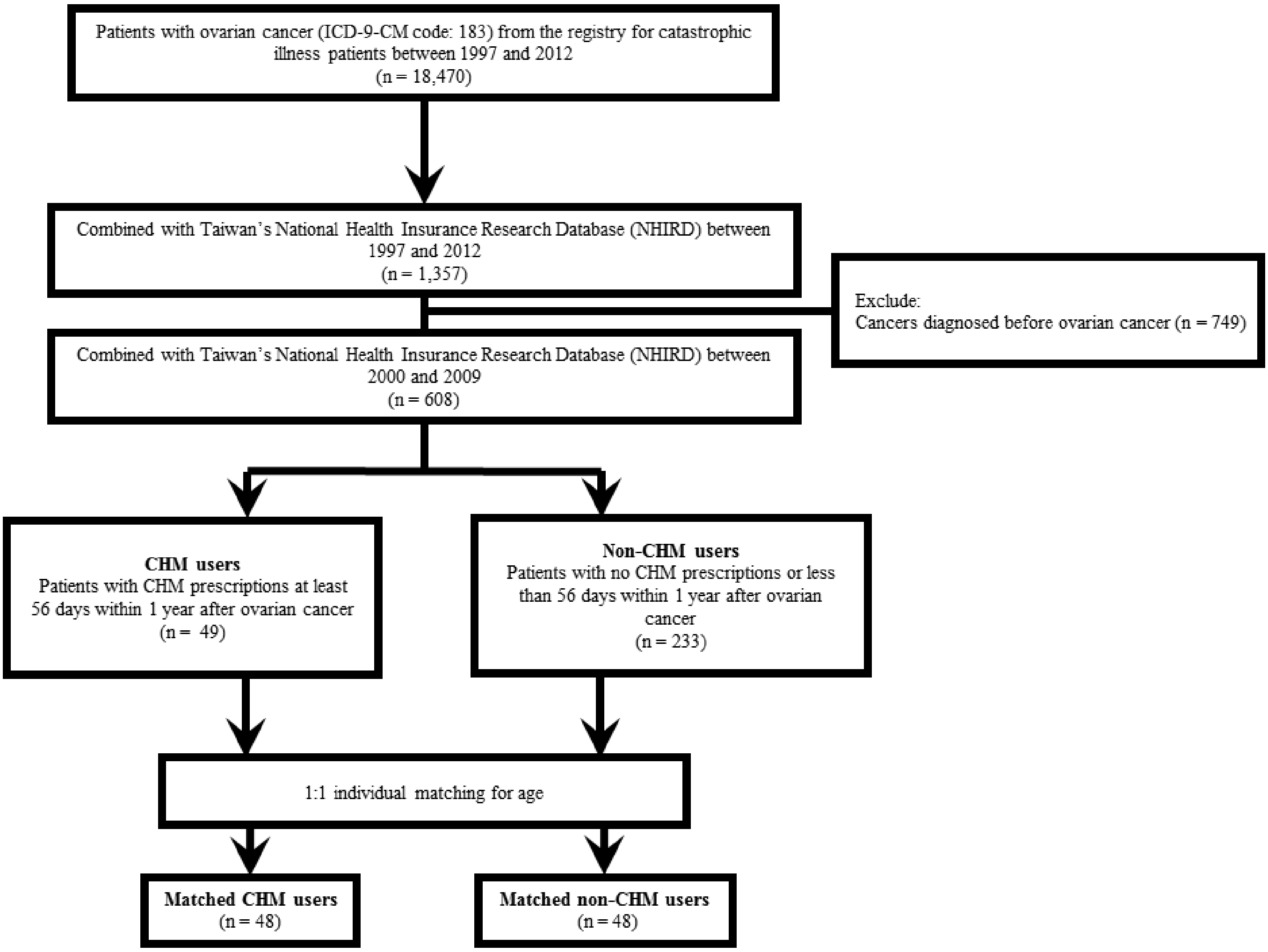
Patients with ovarian cancer (ICD-9-CM code: 183) from the registry for catastrophic illness patients between 1997 and 2012
(n = 18,470)
Combined with Taiwan's National Health Insurance Research Database (NHIRD) between 1997 and 2012
(n = 1,357)
Exclude:
Cancers diagnosed before ovarian cancer (n = 749)
Combined with Taiwan's National Health Insurance Research Database (NHIRD) between 2000 and 2009
(n = 608)
CHM users
Patients with CHM prescriptions at least 56 days within 1 year after ovarian cancer
(n = 49)
Non-CHM users
Patients with no CHM prescriptions or less than 56 days within 1 year after ovarian cancer
(n = 233)
1:1 individual matching for age
Matched CHM users
(n = 48)
Matched non-CHM users
(n = 48)

Figure S3

A

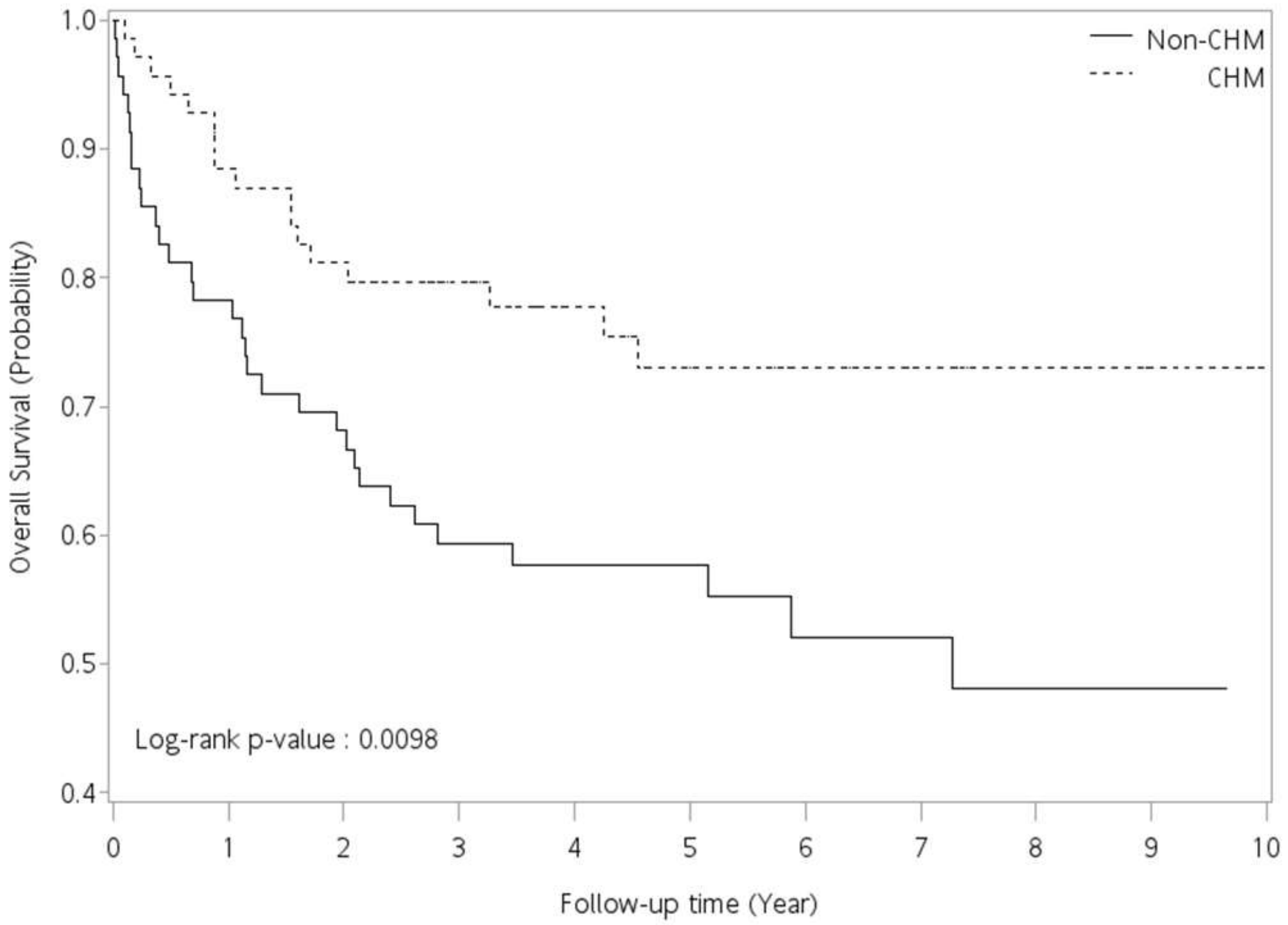

Non-CHM
CHM
Overall Survival (Probability)
1.0
0.9
0.8
0.7
0.6
0.5
0.4
Log-rank p-value : 0.0098
0
1
2
3
4
5
6
7
8
9
10
Follow-up time (Year)

Figure S3

B

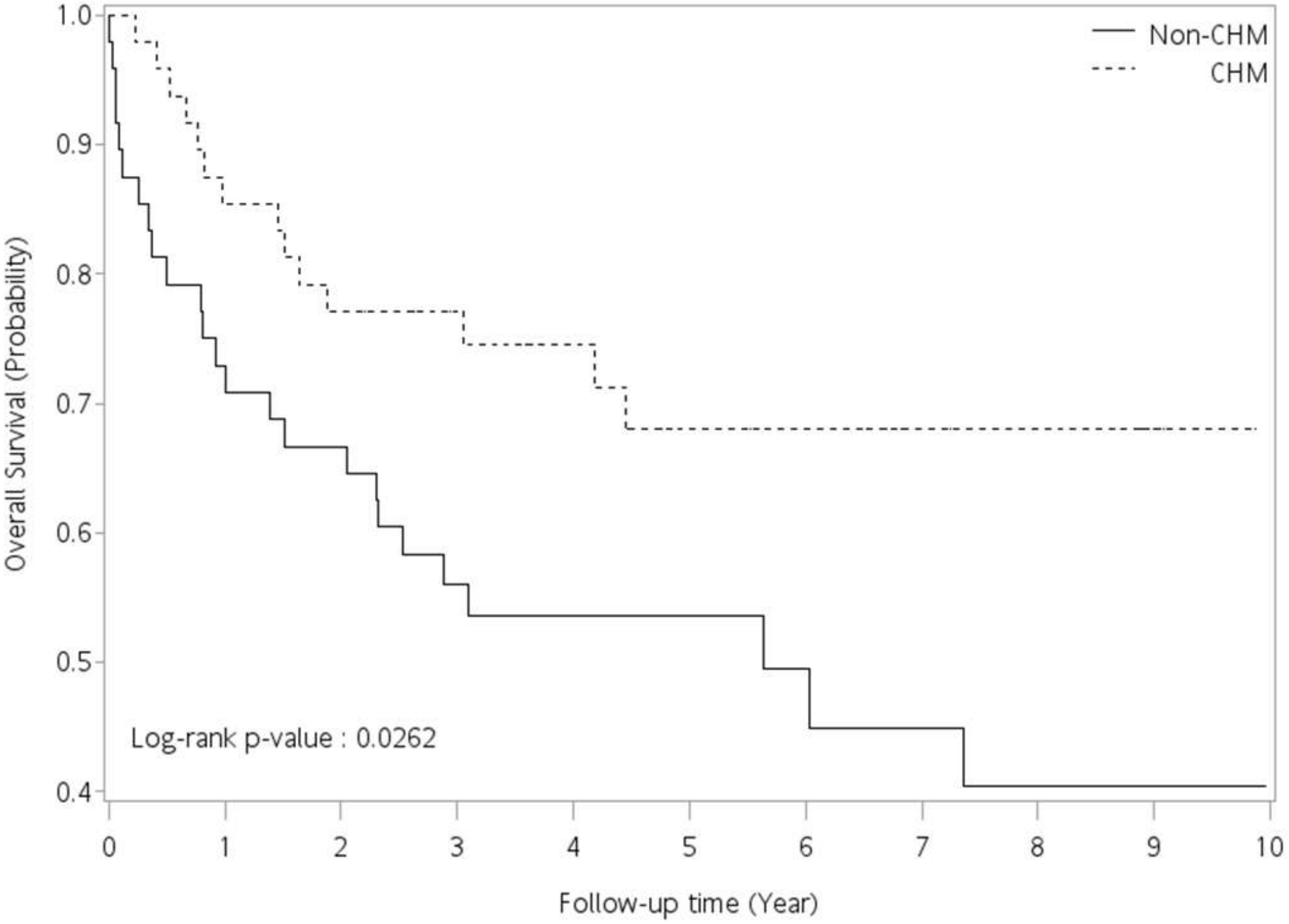

Non-CHM
CHM
Overall Survival (Probability)
1.0
0.9
0.8
0.7
0.6
0.5
0.4
Log-rank p-value : 0.0262
0
1
2
3
4
5
6
7
8
9
10
Follow-up time (Year)
